# 

**Published:** 1892-06-04

**Authors:** 


WITH EXTRA NURSING SUPPLEMENT
Per Annum, 8s. 6d., post free.
Monthly Parts, 6d.; post free, 8d.
Ditto per Annum, 8s., post free.

### THE HOSPITAL

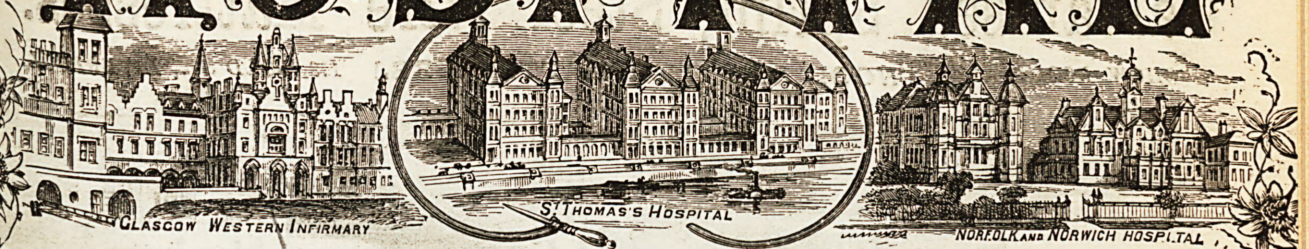

WITH EXTRA NURSING SUPPLEMENT.

No. 297. Vol. XII.] SATURDAY, JUNE 4th, 1892. [TWOPENCE.

